# The evolving role of health care aides in the long-term care and home and community care sectors in Canada

**DOI:** 10.1186/1478-4491-11-25

**Published:** 2013-06-14

**Authors:** Whitney Berta, Audrey Laporte, Raisa Deber, Andrea Baumann, Brenda Gamble

**Affiliations:** 1 Institute of Health Policy, Management & Evaluation, University of Toronto, 155 College Street, Suite 425, Toronto, Ontario, Canada; 2 Institute of Health Policy, Management & Evaluation, University of Toronto, Toronto, Canada; 3 Institute of Health Policy, Management & Evaluation, University of Toronto, Toronto, Canada; 4 School of Nursing, Nursing Health Services Research Unit, McMaster University, Hamilton, Ontario, Canada; 5 Faculty of Health Sciences, University of Ontario Institute of Technology, Oshawa, Ontario, Canada

**Keywords:** Personal support worker, Health care aide, Long-term care, Home and community care, Exponentiation, Substitution, Role boundary, Role expansion, Elderly, Nursing home

## Abstract

Health Care Aides (HCAs) provide up to 80% of the direct care to older Canadians living in long term care facilities, or in their homes. They are an understudied workforce, and calls for health human resources strategies relating to these workers are, we feel, precipitous. First, we need a better understanding of the nature and scope of their work, and of the factors that shape it. Here, we discuss the evolving role of HCAs and the factors that impact how and where they work. The work of HCAs includes role-required behaviors, an increasing array of delegated acts, and extra-role behaviors like emotional support. Role boundaries, particularly instances where some workers over-invest in care beyond expected levels, are identified as one of the biggest concerns among employers of HCAs in the current cost-containment environment. A number of factors significantly impact what these workers do and where they work, including market-level differences, job mobility, and work structure. In Canada, entry into this ‘profession’ is increasingly constrained to the Home and Community Care sector, while market-level and work structure differences constrain job mobility to transitions of only the most experienced workers, to the long-term care sector. We note that this is in direct opposition to recent policy initiatives designed to encourage aging at home. Work structure influences what these workers do, and how they work; many HCAs work for three or four different agencies in order to sustain themselves and their families. Expectations with regard to HCA preparation have changed over the past decade in Canada, and training is emerging as a high priority health human resource issue. An increasing emphasis on improving quality of care and measuring performance, and on integrated team-based care delivery, has considerable implications for worker training. New models of care delivery foreshadow a need for management and leadership expertise - these workers have not historically been prepared for leadership roles. We conclude with a brief discussion of the next steps necessary to generating evidence necessary to informing a health human resource strategy relating to the provision of care to older Canadians.

## Background

Health Care Aides (HCAs) are of increasing importance to the delivery of care to older adults living in their own homes [1,2], and in nursing homes [3]. The term HCA is often used synonymously with Personal Support Worker (PSWs), along with other synonymous titles that are health care setting-dependent, including: home support workers, health care aide, hospital attendant, long-term care aide, nurse aide, nursing attendant, patient care aide, psychiatric aide, and resident care aide.

In Canada, HCAs constitute a significant component of the health care labor force, where these workers are concentrated in the long-term care (LTC) and home and community care (HCC) sectors and provide up to 80% of the direct care to elderly Canadians [4]. The exact size of this workforce at the national level is not known; however information is available for some Canadian Provinces through recent initiatives like provincial registries. Approximately 100,000 HCAs deliver care in the most populous Province, Ontario (13.5 million), where 57,000 work in long-term care facilities and 34,000 work for home health and social care service providers [4]. In British Columbia, the third most populous Province (4.6 million), over 44,000 HCAs have registered with the British Columbia Care Aide and Community Health Worker Registry since its inception in January 2010 [5]. HCAs are unregulated in Canada, and are not recognized as a profession. In both home and community care (HCC) and long-term care (LTC) settings, HCAs work under the direction of a Registered Nurse (RN) or Registered Practical Nurse (RPN).

As the costs of health care escalate [6], so do the challenges to policy makers and practitioners to provide efficient care without sacrificing effectiveness. One notable response to cost containment pressures relating to the delivery of care for older Canadians has been the introduction of new policies that support aging at home. These policies have effectively shifted care from ostensibly more costly acute care settings to the HCC and LTC sectors [7]. The consequent increase in demand for HCAs has been amplified by the concurrent trend to substitute higher cost regulated workers, like nurses, with lower cost unregulated workers [8-10].

As recently as a decade ago, the role of HCAs in Canada was a purely supportive one involving assistance with daily living activities (ADLs), such as bathing, dressing, meal preparation and other ‘light’ household tasks. In the current environment, elderly clients living in their homes, and residents receiving care in nursing homes, require increasingly complex care [11]. The shifts in the workforce that have led to substitution have also led to role expansion, where the roles of some HCAs now include delegated acts for things such as catheterization and injection. Role expansion among these unregulated workers has prompted calls for standardized approaches to preparation/training, and to supervision [4].

Increasingly, a number of jurisdictions in Canada have encountered recruitment and retention challenges relating to these increasingly important caregivers [12]. Keefe and colleagues have recently sought to initiate research and discourse to develop human resource strategies relating to these workers [12]. These are admirable efforts that underscore the importance to elder care of understanding this understudied workforce [13-15] about which we ‘know’ very little with respect to fairly basic information including the nature of their preparation, their work motivations, their attitudes toward their work, and their aspirations. Work retention, for example, is an acknowledged problem among these workers [1] that we do not yet know how to solve; there are differences in worker preparation and expectations [4,15] with unknown impacts on the quality of care delivered to older Canadians. A first step in developing informed human resources strategies, then, must be to endeavor to learn more about these workers. Specifically, we need to know more about the roles of HCAs in direct care to older Canadians; we need to know more about how and where they work; we need to gain an understanding of their work motivations, attitudes and aspirations; and we need to have a more comprehensive understanding of the variation, nature, and adequacy of their preparation. This can inform the development of recruitment programs, incentive systems, and retention and training strategies for these workers in the future.

The discussion that follows is one step toward addressing these knowledge gaps, and serves as a call for further work that stands to inform future human resource planning efforts relating to the delivery of care to older Canadians. We share and elaborate insights, regarding these workers and their work, which emerged from a focus group discussion that we recently led among industry experts actively engaged in long-term care delivery and policy decision making in Canada.

## Methods

Ethics approval for this study was obtained through the University of Toronto’s Ethics Review Board.

### Participants

Six industry experts participated in a focus group. Three individuals were in high-level management positions with three organizations that represent HCAs, including PSWs, in Ontario (2), and nationally (1); one individual was an executive with an entity that represents organizations employing HCAs/PSWs in the HCC sector; and two participants were from two associations representing for-profit (1) and not-for profit (1) employer organizations of PSWs/HCAs in the long-term institutional care sector. All of the organizations represented by our focus group participants, and the participants themselves, were intentionally selected in the interests of gaining a comprehensive understanding of the nature, scope and challenges inherent in the work currently done by personal support workers and health care aides in the LTC and HCC sectors. Of note, the scope of five of the six organizations represented by focus group participants extended beyond the sectors of immediate interest to us (that is, LTC and HCC) and beyond the health population of immediate interest to us (that is, older Canadian persons), to other sectors in which HCAs are employed to care for other populations, including the acute care sector, the rehabilitation sector, complex continuing care, and disabled persons including youth.

### Data collection and analysis

In our two-hour focus group, conducted in February 2012, we sought to better understand the type and scope of work that HCAs do as it relates to caring for older persons in Ontario. We asked focus group participants to describe: the nature of the practice environments for HCAs; how the work of HCAs is structured; and how/if their roles in the delivery of care in the residential long-term care and home and community care sectors has changed since the mid-1990s. The focus group was tape recorded and transcribed verbatim by a professional transcriptionist contracted by the principal investigators; the transcriptionist removed any information identifying the participants. Two of the investigators reviewed the transcript and identified preliminary themes that emerged from the transcript data [16]. A draft of this initial analysis and anonymized copies of the focus group transcript was then distributed to all project team members for review. Key themes, and passages illustrative of them, were subsequently discussed in an all-team meeting; this resulted in reassigning one passage to a different theme. No new themes were identified as a consequence of this discussion. As a further effort to ensure interpretive validity [17], a revised draft of the analysis was developed and distributed to all six focus group participants for their review. All focus group participants were asked to offer their feedback and reflections regarding the accuracy and adequacy of data coding and of the key themes that were identified by the project team. Modest revisions to the theme labels were made, and undertaken to produce the final version of the findings/analysis offered below. To an extent, internal validity regarding the causal inferences/relationships that emerged from our analysis was assured through revisiting some of the literature on these dynamics, cited above, that motivated this research [18].

## Discussion

### What HCAs do

Organizational behavior theorists and practitioners distinguish between the behaviors required or expected of workers, and those demonstrated outside of role expectations. This distinction is relevant to the work that HCAs do. *Role-required* behaviors include ADLs that are the assumed purview of these workers in both the HCC and LTC sector [4]. There is an increasing reliance on HCAs in the HCC sector, in particular, to perform delegated acts for regulated health professionals, and a concurrent addition of more instrumental activities of daily living (IADLs) to their service repertoires. While ADLs include bathing, toileting and personal care, IADLs include transportation, meals, and social activities. Our expert informants referred to the role expansion of HCAs as ‘exponentiation’; in Canada, the retirement of ‘heavily-credentialed’ baby boomers, coupled with cost containment pressures, has led to their replacement with less- or non-credentialed health care workers that are less costly and faster to produce.

Increasingly, HCAs are relied upon for *extra-role behaviors*. Extra-role behaviors include emotional support, and other behaviors beyond those prescribed by a role. While they have not been studied in the LTC or HCC contexts, extra-role behaviors have been found to positively influence performance outcomes in other health care settings [19] and in other industries [20]. One expert offered these compelling examples of extra-role behaviors by way of illustrating how these workers influence the quality of life of their elderly clients:

‘We have PSWs [HCAs] who will take a late shift to see a client because they know the shift isn't long enough to provide the care that's required…beyond the time that they’re paid just to make sure that that person is adequately cared for. There are PSWs [HCAs] who take home the seniors’ laundry and do it on a regular basis…We want people to go above and beyond. There are lots of people in the hospital and in long-term care that go above and beyond. We know that people do this because it's a human service industry…’ (IE1)

Related to extra-role behaviors, role boundaries are described as one of the biggest concerns, currently, among employers of HCAs in the current cost-containment environment. Role boundaries refer to the relationships that HCAs maintain with their clients, where some care givers may exceed their ‘role boundaries’, and over-invest in care beyond levels expected – or desired – on the part of their employers. Role boundaries are important for workers to understand in order to ensure that they are expending their time effectively and efficiently. However, HCAs who are engaged in enduring relationships with their clients may find it difficult to distinguish between role, extra-role, and ‘excessive’ extra-role behaviors due to their strong feelings of obligation, concern, and affection for their clients.

### Factors that influence what HCAs do

A number of factors significantly impact what these workers do, including market-level differences, job mobility, and work structure. Market-level differences include differences in barriers to entry, with lower barriers to entry for HCAs to the HCC sector, than for the LTC sector since the latter must obtain certification in many Canadian jurisdictions. On the other hand, wages for HCAs in HCC are generally lower than in the LTC sector, and the disparity is aggravated when HCAs bear the costs for travel to and between their clients.

Job mobility is limited among these workers. The LTC sector is viewed as the ‘employer of choice’ where the most experienced and better-trained workers in the HCC sector transition to LTC - because they can. Retention of HCAs in the HCC sector, then, is a problem. In some jurisdictions, this problem has been magnified by changes to the regulations that limit transfers to those that are deemed to have acquired ‘equivalent’ experience to workers in LTC - criteria that favor the transfer of the most highly qualified workers from the HCC to the LTC sector. Without a coherent systems-level human resources strategy, this inter-sectoral ‘cannibalism’ will persist and merits immediate attention, since transfers out of the HCC sector effectively undermine recent policy initiatives put in place to sustain older Canadians in their homes. By way of further underscoring the importance of adopting a systems thinking approach to human resource planning, we know that changes to the legislation in one province can constrain worker choice in other provinces: new, more stringent transfer requirements in Ontario, for example, stand to impact approximately 1,000 out-of-province workers who will now face higher barriers to entry into that province’s LTC workforce, and will be limited to (starting in) the HCC sector.

Work structure influences what these workers do, and how they work. For instance, the demand for HCAs’ services in the HCC sector tends to be concentrated over early morning hours and late at night many HCAs in the HCC sector work for three or four different agencies in order to accumulate hours that equate to full-time employment. In Canada, there is considerable geographic variation in the availability of jobs for these workers such that in some jurisdictions - particularly rural areas and in the less densely populated Northern parts areas - these workers simply cannot rely solely on work as a HCA, and must supplement with other types of work. While there is the general sense that there is greater autonomy in the work of HCAs in the HCC sector, other features of this work (described above) still render work in the LTC sector generally more attractive. That said, the fact that work in either of these sectors is less well remunerated than similar work in other sectors, such as the acute care sector [21], may outweigh the attractiveness of greater autonomy.

### HCA role preparation

Expectations with regard to HCA preparation have changed over the past decade in Canada, and worker preparation was identified by our expert informants as a high priority health human resource issue. There is an increasing emphasis on improving quality of care and measuring performance in the HCC and LTC sectors that has led to higher expectations in terms of the *competencies and abilitie*s of these workers. There is a *new emphasis* on *critical thinking* and provision of *decision support* to facilitate on-the-spot, evidence-based responses to care challenges. It is anticipated that future emphasis will be placed on prevention and promotion, and the ‘upstream’ management of chronic diseases on the part of these workers. Our experts remarked that variation in terms of worker preparation, and the bimodal distribution of this workforce, is likely to present challenges to preparing workers to meet these changing expectations:

‘…because you have successive groups of PSWs [HCAs] that have been grandfathered-in under various pieces of legislation. So, you have people with varying degrees of formal training and a lot of experience and the ones who have the most education are actually part-time, flex-time, you know and so you then have this mix of people and you need to value experience but you also need to value current practice and right now, we're not really thinking of these workers…as knowledge workers that need to maintain their practice…and that need to be up to date. We don't have ways of maintaining skills that are cost effective…’ (IE6)

An emerging movement to deliver care through inter-professional collaborative models suggests a need to reconfigure the training and education of HCAs since most of these workers have not been trained to work within a team care delivery structure. While HCAs must now obtain certification in some Canadian jurisdictions, there is *persistent variation* in the *level of preparation* among HCAs employed in the HCC sector, in particular. There is no stipulated minimum standard of preparation, and while some employers offer training, others do not. HCAs in the HCC sector can avail themselves of training offered by educational institutions through ‘approved’ preparatory programs, and/or some employer agencies offer them in-house training opportunities. However in some parts of Canada, access to approved preparation programs is difficult due to geographic distances.

Beyond differences in the level of preparation, there are differences in the *mode of worker preparation*; recently-trained HCAs have been exposed to theory-based preparation as compared to a historical reliance on hands-on experiential learning. Reliance on more didactic approaches permits faster production of more of these workers, where experiential learning requires more time and resources. The impact of this shift on the quality of care for older Canadians, and the optimal mode(s) of preparing these workers in light of their expanding roles, are important questions to examine.

We referred earlier to recent changes in how care is delivered to older Canadians, and to the phenomenon of exponentiation. Both foreshadow an emerging need for *management expertise* in the LTC and HCC sectors. Increasing numbers of these workers with no increases in regulated professionals will necessitate different management and accountability structures to effectively manage and allocate workers’ time, and to ensure the currency of skills and knowledge. HCA-led management hierarchies, of other HCAs, are relatively novel to these sectors [22]. These workers have not traditionally been in leadership roles, and structures have historically been flat with few career advancement opportunities.

## Conclusions

The role of HCAs in Canada is evolving. A new reliance upon integrated team-based care delivery, increasing reliance upon these workers to undertake delegated tasks, demand for increasingly complex care, and the need to increasingly rely upon these workers to think critically and execute real-time, evidence-based care decisions - all point to the need to re-conceptualize HCAs as *knowledge workers* and to develop initiatives that equip them to work in this capacity. While we have referred to myriad facts and factors that shape the nature and scope of their work, and stand to further impact their work in the future, we do not know ‘who these workers are’. That is, we know little about the attitudes of these workers toward change, their attitudes toward their work generally, their work motivations, and the relationships between work attitudes and outcomes. We know nothing about the empirical relationships between modes of preparation, and quality of care decision-making, and what the moderating variables (for example, work attitudes) might be. We do know that these are important things *to* know [3]: decades of research in organizational behavior in other industries [23], and more recent work in other health care settings focusing on other types of workers [19,24,25], demonstrates the relationships between work attitudes and work outcomes, and their importance to decision making quality, and to work behaviors such as turnover. Beyond our ‘need to know’, a systems perspective when formulating human resources strategy is needed to address the broad issues of supply, demand, and resource interdependencies across health care sectors that can influence the provision of care to older Canadians [26].

## Competing interests

The authors declare that they have no competing interests.

## Authors’ contributions

WB and AL led in the conceptualization of the study. All authors participated in the data collection; WB and AL undertook the initial thematic analysis of the focus group transcript data; all authors contributed substantively to finalizing the coding scheme through iterative discussion of the data in light of the initial thematic analysis. WB drafted the first version of the manuscript; AL, RD, AB and BG contributed substantively to subsequent revisions to the manuscript. All authors have given final approval of the current version of the manuscript.
